# Inactivation of nucleolin leads to nucleolar disruption, cell cycle arrest and defects in centrosome duplication

**DOI:** 10.1186/1471-2199-8-66

**Published:** 2007-08-10

**Authors:** Iva Ugrinova, Karine Monier, Corinne Ivaldi, Marc Thiry, Sébastien Storck, Fabien Mongelard, Philippe Bouvet

**Affiliations:** 1 Laboratory Joliot-Curie, CNRS USR 3010, University of Lyon, Ecole Normale Supérieure de Lyon, 46 Allée d'Italie, Lyon, France; 2 Laboratory of molecular biology of the cell, CNRS UMR 5239, IFR128 Biosciences, University of Lyon, Ecole Normale Supérieure de Lyon, 46 Allée d'Italie, Lyon, France; 3 Laboratory of Cell Biology, Department of Life Sciences, Faculty of Sciences, University of Liege, Liege, Belgium

## Abstract

**Background:**

Nucleolin is a major component of the nucleolus, but is also found in other cell compartments. This protein is involved in various aspects of ribosome biogenesis from transcription regulation to the assembly of pre-ribosomal particles; however, many reports suggest that it could also play an important role in non nucleolar functions. To explore nucleolin function in cell proliferation and cell cycle regulation we used siRNA to down regulate the expression of nucleolin.

**Results:**

We found that, in addition to the expected effects on pre-ribosomal RNA accumulation and nucleolar structure, the absence of nucleolin results in a cell growth arrest, accumulation in G2, and an increase of apoptosis. Numerous nuclear alterations, including the presence of micronuclei, multiple nuclei or large nuclei are also observed. In addition, a large number of mitotic cells showed a defect in the control of centrosome duplication, as indicated by the presence of more than 2 centrosomes per cell associated with a multipolar spindle structure in the absence of nucleolin. This phenotype is very similar to that obtained with the inactivation of another nucleolar protein, B23.

**Conclusion:**

Our findings uncovered a new role for nucleolin in cell division, and highlight the importance of nucleolar proteins for centrosome duplication.

## Background

There is increasing evidence that nucleoli play important roles in the regulation of many fundamental cellular processes, including cell cycle regulation, apoptosis, telomerase production, RNA processing, monitoring and response to cellular stress [1-3]. These multiple functions can be achieved by the transient localization of several hundred proteins within the nucleolar structure [4]. In addition, several well known nucleolar proteins seem to have multiple functions. Amongst them, the abundant nucleolar proteins nucleophosmin (B23) and nucleolin (C23) have been the subject of numerous studies.

Although nucleophosmin and nucleolin do not share any structural homology, they seem to have numerous functions in common. The predominant location of these proteins in the nucleolus strongly suggested that they could be involved in ribosome biogenesis. Both proteins interact with nucleic acids [5,6], and could be viewed as ribosome assembly factors. Despite the fact that endoribonuclease and molecular chaperone activities have been reported for nucleophosmin [7-9], its contribution to ribosome biogenesis is still unclear. The function of nucleolin in ribosome biogenesis has been well documented [10,11]. Its interaction with ribosomal proteins [12] and with specific pre-rRNA sequences [6,13,14], and its implication in the first step of pre-rRNA maturation [15], also suggest that nucleolin could be an important ribosome assembly factor. In addition, nucleolin seems to be involved in the control of pre-rRNA accumulation [16-18].

Several observations suggest that nucleolin is also a major actor in promoting cell proliferation. Nucleolin levels are higher in tumours and actively dividing cells [19-23], and are widely used as a marker of cell proliferation. Furthermore, overexpression of nucleolin cooperates with oncogenic mutant Ras in a rat embryonic fibroblast transformation assay [24].

Nucleolin seems to be able to affect ribosomal RNA gene expression in different ways. A strong link between nucleolin phosphorylation, proteolysis and the production of ribosomal RNA has been observed [25-27]. The inhibition of nucleolin proteolysis leads to lower rRNA transcription in an *in vitro *transcription system [27], while the injection of nucleolin antiserum leads to 2–3.5 fold stimulation of pre-rRNA synthesis in *Chironomus tentans *salivary glands [28]. Injection of a 2–4 fold excess of nucleolin in *Xenopus laevis *stage IV oocytes also leads to a significant reduction in the accumulation of 40 S pre-RNA [17]. It has also been demonstrated that nucleolin phosphorylation can be triggered by a variety of stimuli such as androgens and growth factors [29-33], and that this phosphorylation is invariably accompanied by increased rRNA transcription and cell proliferation.

In addition to nucleolar functions, nucleophosmin and nucleolin seems to share additional functions. Both proteins are histone chaperones [34,35], and appear to be involved in different aspects of DNA metabolism and chromatin regulation [36,37].

Nucleophosmin is also directly implicated in cancer pathogenesis, as the NPM1 gene is found mutated and rearranged in a number of haematological disorders [38]. Nucleophosmin is essential for mouse embryonic development, as the inactivation of the Npm1 gene leads to unrestricted centrosome duplication and genomic instability [39]. It has been proposed that during mitosis, nucleophosmin associates with duplicated centrosomes and prevents their reduplication until the G1/S phase, when it dissociates from them after phosphorylation by Cdk2/E [40,41]. Wang et al. further showed that the centrosomal association of nucleophosmin relies on the Ran-Crm1 complex [42]. Although nucleolin has not previously been shown to be involved in the regulation of centrosome duplication, it is interesting to note that nucleophosmin interacts directly with nucleolin [43], and that a nucleolin-like protein, together with other nucleolar proteins, has been found associated with centrosomal structure [44].

To determine the function of nucleolin in cell proliferation and cell cycle regulation we used siRNA to knock down nucleolin expression. We found that nucleolin is not only required for cell proliferation, but that its down regulation also leads to unrestricted centrosome duplication, as described for the inactivation of nucleophosmin.

## Results

### Nucleolin is required for nucleolar structure formation

**Figure 1:**
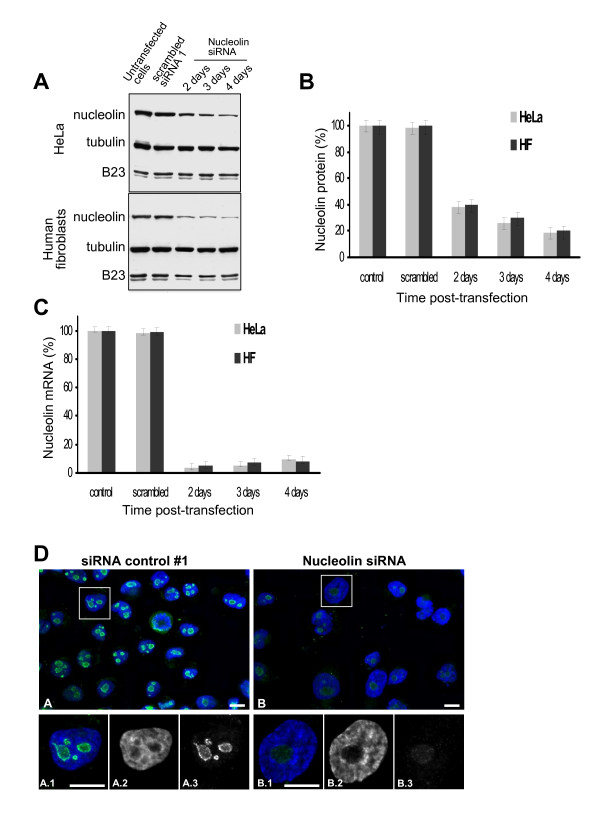
**siRNA – mediated down regulation of nucleolin**. A. Western blots of nucleolin protein in untransfected control cells, mock (transfected with siRNA control #1) and siRNA against nucleolin (mix siRNA #2 and #4) treated cells. At the indicated times, HeLa and human primary fibroblast cells were harvested and protein extracts were analyzed by Western blotting with anti-nucleolin antibody. Equal loading was verified using anti-tubulin antibodies. The amount of B23 protein was also analyzed using anti-B23 antibodies. B. Quantification of the Western blots. Data were normalized to the tubulin protein. The normalized nucleolin protein level was set to 100% in control cells. Data are from three independent experiments. C. Quantitative RT-PCR. At indicated times after transfection, HeLa and human primary fibroblast cells were harvested, total RNA was isolated and used for cDNA synthesis and quantitative PCR with nucleolin or cytoplasmic β-actin specific primers. Data were normalized to the amount of β-actin mRNA. Untransfected control and scrambled (transfected with scrambled siRNA) cells were also used and all data were normalized to the amount of nucleolin mRNA in control cells. Data are from three independent experiments. D. Immunofluorescence analysis. Four days after siRNA transfection (control scrambled siRNA #1, panels A, or mix of siRNA #2 and #4 panels B), HeLa cells were examined by immunofluorescence using anti-nucleolin antibody in green. DNA was counterstained with DAPI (Blue). In panels A.1 to A.3 and B.1 to B.3, a zoom on one representative cell is shown to highlight the modification of the shape and number of nucleolar structures after nucleolin depletion. Bars represent 10 μm.

Nucleolin is highly expressed in proliferating cells, and is believed to be important for cell growth. In order to study the role of nucleolin in cell proliferation, we tested different siRNA for their ability to significantly decrease the amount of nucleolin in human cells. Preliminary experiments with 4 different siRNA sequences directed against distinct domains of nucleolin and 2 siRNA controls (See Additional file 1) were performed and allowed us to select the best siRNA duplexes able to induce a significant decrease of nucleolin expression. Of these 4 siRNA, two significantly reduced nucleolin protein levels (#2 and #4, see Additional file 2). Neither one of the 2 control siRNA was able to significantly reduce the level of nucleolin protein. A mixture of siRNA #2 and #4 were transfected in HeLa cells or in a primary culture of human fibroblast, followed by time course kinetics of nucleolin expression inhibition (Figure 1A, B). In both HeLa cells and human primary fibroblasts, the level of nucleolin protein started to decrease 2 days after siRNA transfection to reach a minimum after 4–5 days, corresponding to a decrease of about 80% (Figure 1B). Interestingly, the level of B23 (Figure 1A) and fibrillarin (data not shown), other abundant nucleolar proteins, was not affected by the decreased nucleolin expression. This lower level of protein expression was perfectly correlated with a decrease in nucleolin coding mRNA (Figure 1C). Nucleolin mRNA levels remained low for at least 4 days. The decrease in nucleolin protein levels was confirmed by immunofluorescence analysis of HeLa cells transfected with nucleolin siRNA (mix of #2 and #4) (Figure 1D, panel B) compared to control cells transfected with the scrambled siRNA (#1). In addition, these microscopic observations also indicated an important morphological modification of nuclei that expressed low levels of nucleolin. These nuclei appeared bigger and more rounded with a single large nucleolar body (Figure 1D, panels B1 to B3) compared to cells transfected with the siRNA control #1 (Figure 1D, panel A).

**Figure 2:**
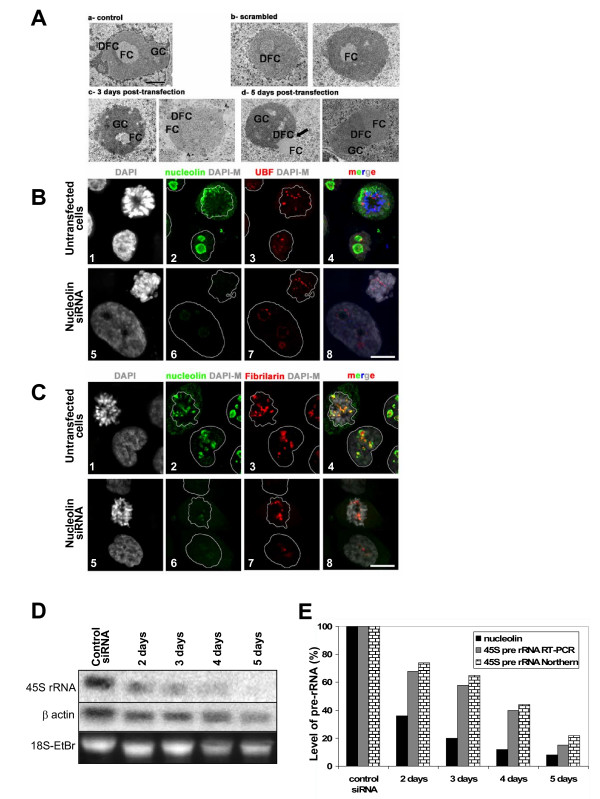
**The nucleolar structure is disrupted in nucleolin depleted cells**. A. Electron micrographs of nucleoli in control untransfected cells (a), mock transfected cells with scrambled siRNA (b) and cells transfected with nucleolin mix siRNA #2 and #4 (c, d). Cells were fixed 3 days (a, c) or 5 days after transfection (b and d). FC: fibrillar center; DFC: dense fibrillar component; GC: granular component. Bar represents 1 μm. B and C. Co-detection of nucleolin (green) and UBF (B, red) or fibrillarin (C, red) in HeLa cells transfected with control siRNA or with nucleolin mix siRNA #2 and #4, 5 days after transfection. Images of DNA counterstaining by DAPI are also shown. Images correspond to a projection of several deconvolved sections acquired every 0.2 μm. An interphase nucleus and a mitotic cell are shown in each panel. Bars represent 10 μm. D and E. Accumulation of pre-ribosomal RNA in HeLa cells transfected with control siRNA or with nucleolin mix siRNA #2 and #4. Total RNA was extracted at different days after transfection and used for northern blot analysis (D) or quantitative RT-PCR (E). For the northen blot, an oligonucleotide complementary to the 5'ETS was used as a probe. The blot was also hybridized with a probe specific for β actin to normalize the data. Ethidium bromide staining (18S-EtBr) of the gel before transfer is also shown to show RNA integrity and equal loading of RNA (E). The normalized data of the northern blots are shown, together with the data of the quantitative RT-PCR, and with the amount of nucleolin protein present in these cells as determined by nucleolin western blot (not shown).

Since nucleolin is a major protein of the nucleolus, one might expect that depletion of nucleolin would affect nucleolar structure. As seen by electron microcopy, the nucleolus consists of three main components, designated fibrillar centers (FC), dense fibrillar components (DFC), and granular components (GC). Ribosomal RNA genes are localized in the FC, pre-rRNA resides in the DFC, while late processing steps of ribosome biogenesis are found in GC. In untransfected control and in scrambled siRNA transfected cells, FC is found in the center of the nucleolar structure, surrounded by the DFC. The DFC is in turn surrounded by GC, which fills out the peripheral parts of the nucleolus (see Figure 2A, a-untransfected control). Nucleolin is usually found in the DFC and GC of nucleoli, UBF (Upstream Binding Factor) in the FC, and fibrillarin in the DFC. Electron microscopy analysis of control and siRNA transfected cells revealed a profound modification of the different nucleolar compartments in absence of nucleolin (Figure 2A, panels c and d), associated with an apparent increase of the nucleolar volume. Fibrillar centers and dense fibrillar centers were observed at the periphery of the granular component, and a complete segregation of these compartments could even be observed at later time points following transfection (Figure 2A, panel d). This reorganization of the nucleolar compartment was confirmed by immunofluorescence analysis of nucleolin, UBF and fibrillarin in cells transfected with a control scrambled siRNA, and in nucleolin depleted cells (Figure 2B, C). In interphase control cells, UBF was mainly present in foci that probably correspond to the internally localized FC, whereas nucleolin labeled nucleolar peripheric structures corresponding to DFC and GC. Interestingly, in nucleolin depleted cells, UBF no longer appeared as foci, but as ring-like structures surrounding the DAPI depleted regions (Figure 2B). This staining could correspond to the delocalized FC observed by electron microscopy (Figure 2A, c–d). In contrast, the localization of fibrillarin does not seem to be affected in nucleolin depleted HeLa cells (Figure 2C). This segregation of nucleolar compartments was also found when cells were treated with low amounts of actinomycin D, an inhibitor of polymerase I transcription [45-47]. To determine if the lower expression of nucleolin led to a lower accumulation of pre-ribosomal RNA, cells were collected at different times after siRNA transfection, and the 45S pre-rRNA analyzed by northern blot (Figure 2D) or quantitative RT-PCR (Figure 2E). Using these two techniques, we found that nucleolin depletion leads to a 5–6 fold reduction of 45S accumulation, as expected [16-18].

Nucleolin has been implicated *in vitro *in the first processing step of pre-rRNA [15]. It was therefore interesting to determine if down-regulation of nucleolin was able to modify the efficiency of this processing step *in vivo*. Analysis of the efficacy of this first processing step in HeLa cells did not allow us to detect significant changes in the level of cleavage within the 5'ETS upon nucleolin depletion (data not shown). In addition, the general pattern of pre-rRNA processing did not seem to be altered. The low level of remaining nucleolin might be sufficient to allow for correct processing. Furthermore, since this primary processing step within the 5'ETS cleavage is not complete in these cells, and it is not known how it is regulated when polymerase I transcription is inhibited, it might be difficult in this experimental system, to detect the effect of nucleolin depletion on this cleavage.

### Nucleolin regulates cell proliferation and cell cycle progression

**Figure 3:**
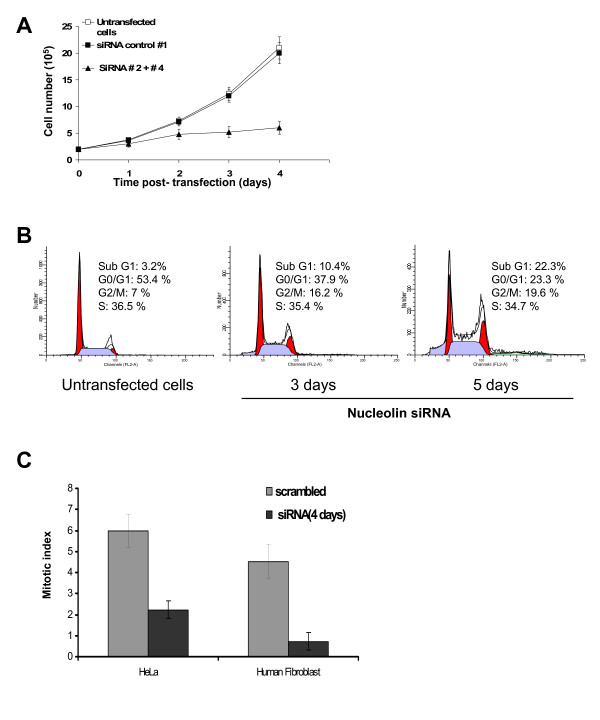
**Nucleolin depletion leads to decreased cell growth and G2/M accumulation**. A. Growth curves of HeLa cells, untransfected or transfected with control siRNA # 1 or the siRNA mix #2 and #4. Cells were counted at the indicated time after transfection. The graph represents the average of 5 independent experiments. B. Analysis of cell cycle in nucleolin depleted cells. Untransfected and transfected HeLa cells were stained with propidium iodide and processed for FACS analysis. Histograms from a representative experiment showing G1 and G2/M populations shaded in red and S phase population in blue. Sub G1 represent apoptotic cell population. The vertical and the horizontal axes represent respectively cell number and DNA fluorescence intensity. The percentages of cells in individual cell cycle phases were quantified with ModFit LD software. C. Mitotic index of HeLa cells and human primary fibroblasts transfected with control siRNA or the siRNA mix #2 and #4. After 4 days of transfection, cell populations were stained with an anti phospho S^28^-H3 antibody and cyclin B1 to detect mitotic cells. Positive cells were scored by microscopic observation. This graph represents the average of the different experiments (DAPI, PH3S28 and cyclin B1) shown in additional file 5.

To determine if nucleolin depletion affects cell proliferation, the growth rate of these cells was measured and compared to that of cells transfected with scrambled siRNA and untransfected control cells. This was achieved by counting cells at different time points after transfection of siRNA #2 and #4 (Figure 3A). As soon as the level of nucleolin protein decreased (second day after transfection, see Figure 1B), the number of cells increased more slowly and was almost steady compared to untransfected cells or to cells transfected with a scrambled siRNA, suggesting that cell growth was affected by low nucleolin amount. To determine if cell growth was blocked at a specific phase of the cell cycle, flow cytometry analysis (FACS) was performed on siRNA transfected and control HeLa cells (Figure 3B). Transfection of control siRNA did not change the cell cycle profile of HeLa cells (Additional file 3), whereas transfection of the mix of siRNA #2 and #4 significantly altered the cell cycle (Figure 3B). During nucleolin time-course depletion, cell cycle analysis showed a progressive and significant decrease in the cell population with a 2n DNA content (G1: 53% in untranfected population to 37.9% in population transfected for 3 days to 23% in population tranfected for 5 days, Figure 3B), whereas a progressive and significant increase was observed in the population with a 4n DNA content (G2/M: 7% in untranfected population to 16.2% in population transfected for 3 days to 19% in population tranfected for 5 days, Figure 3B). Based on this cell cycle analysis, the number of cells in S phase was approximately unchanged (36.5% to 35.4% to 34.7%, Figure 3B). The stability of the S-phase population upon nucleolin depletion was confirmed by manually counting cells exhibiting a positive BrdU staining under the microscope (Additional file 4). Similar trends were observed when cell cycle progression was analyzed on primary fibroblast cultures transfected with control siRNA or with a mix of siRNA #2 and 4 directed against nucleolin (data not shown). siRNA duplexes that have no effect on nucleolin protein levels (#1 and #3) also have no effect on cell cycle progression (see Additional file 3), further showing that the observed cell cycle effects are the consequence of the specific depletion of nucleolin.

To determine if the absence of nucleolin was able to affect mitotic progression, we measured the effect of nucleolin depletion on the number of cells in mitosis (Figure 3C and Additional file 5). HeLa and fibroblast cells were transfected with control or anti-nucleolin siRNA, and cultured for 4 days before fixation and staining with DAPI, and immuno-detection of either phosphorylated serine 28 of histone H3 (PH3-S28), or cyclin B1, to identify mitotic cells. PH3-S28 labeling and DAPI staining showed a significant decrease in mitotic cells (about 65% decrease in HeLa and 90% decrease in fibroblast, Additional file 5). The decrease in the mitotic index upon nucleolin depletion was further confirmed by an increase in cyclin B1 staining of chromatin, observed concomitantly with nuclear envelope break down at the beginning of mitosis (Additional file 5). Altogether these results are indicative of a mitotic index reduction (Figure 3C). Therefore, the overall increase in the G2/M population observed by flow cytometry upon nucleolin depletion is not due to an increase in the mitotic population, but is likely to represent an increase in the G2 population. To confirm this hypothesis, immunofluorescence with an antibody against phosphorylated serine 10 of histone H3 (PH3-S10), a G2 marker [48], was performed (Additional file 4). The data show an about 2-fold increase in cells labeled with PH3-S10 upon nucleolin depletion. Therefore, the net gain in cells exhibiting a G2 marker contributes largely to the increased population exhibiting the G2/M DNA content observed upon nucleolin depletion.

As a small number of cells showed polyploidy upon nucleolin depletion (Figure 3B, 5 days), we wanted to ask whether tetraploid cells with a 2n DNA content contribute to the increase in the population size exhibiting a the G2/M DNA content. In situ hybridization performed with a probe specific to the centromere of chromosome 10 on cells transfected with siRNA against nucleolin revealed only very few nuclei with twice the number of hybridization signals observed in control cells (data not shown). This result suggests that tetraploid cells do not significantly contribute to the observed increase in the population exhibiting a G2/M DNA content observed upon nucleolin depletion. Therefore the increase in G2/M population upon nucleolin depletion can be mainly explained by an increase in diploid G2 cells.

**Figure 4:**
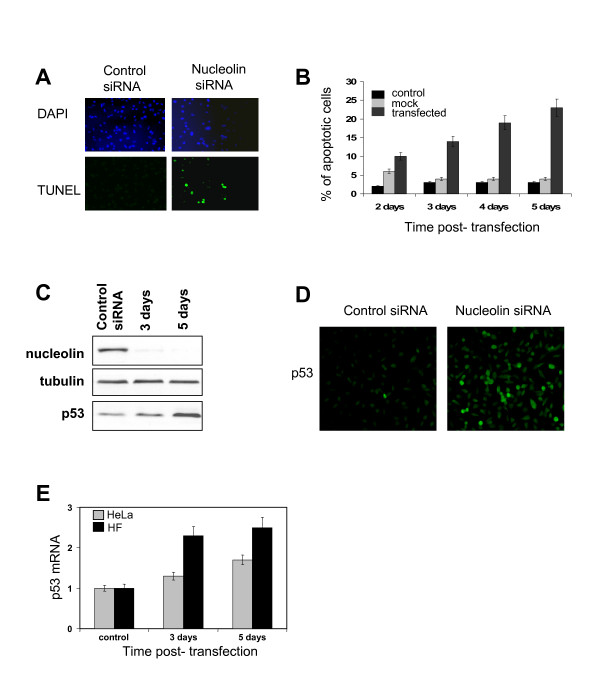
**Nucleolin depletion activates apoptotic pathways**. A. TUNEL staining. HeLa cells transfected with control siRNA # 1 or the nucleolin siRNA mix #2 and #4 were subjected to TUNEL staining 4 days after transfection. B. Quantification of TUNEL-positive cells by counting at least 400 cells in two independent experiments. Error bars indicate ± SD. C. Western blots. At indicated times, protein extracts were analyzed by Western blotting with anti-nucleolin and anti-p53 antibodies. Normalization was performed with tubulin. D. Immunofluorescence of p53 of control or anti-nucleolin siRNA transfected cells 4 days post-transfection. E. Quantitative RT-PCR. Primers to β-actin and p53 were used. Levels of p53 mRNA were calculated against the level of β-actin mRNA as reference gene.

Five days after transfection, about 20% of HeLa cells had a sub-2N DNA content (Sub G1) compared to 3.2% in untransfected control cells (Figure 3B). Consistent with that, tunnel assays (Figure 4A, B) revealed about 22% of apoptotic cells 5 days after transfection. Thus, depletion of nucleolin in HeLa cells triggers apoptosis. Nucleolar disruption in response to cellular stress has been found to stabilize p53 [49]. Indeed, we observed a marked increase in p53 protein levels after depletion of nucleolin (Figure 4C and 4D). This increase may be the consequence of p53 protein stabilization. However, a 2-fold increase of p53 mRNA could also be observed in both HeLa and fibroblast transfected cells (Figure 4E). To determine if apoptosis was responsible for the large reduction in cell growth, observed upon nucleolin depletion (see above) we performed a simulation to remove the contribution of cell death by apoptosis on measured growth curves (see Figure 3A). The growth curve obtained for cells transfected with control siRNA was used as a starting point to calculate the estimated curve (Additional file 6). The percentage of apoptotic cells measured during a 4 day time course (Figure 4B) was subtracted from the total cell population at each time point to remove the apoptotic population from the growing cell population. The reevaluating growth curve was, as expected, below the growth curve obtained for cells transfected with control siRNA, but significantly much higher than the growth curve measured for cells transfected with siRNA against nucleolin (Figure 4B, compare dash curve to plain curves). Our result indicates that in addition to apoptosis induction, nucleolin depletion reduces cell proliferation. Therefore, apoptosis and cell proliferation reduction both act synergistically and ultimately lead to cell growth arrest.

### Inhibition of nucleolin expression induces multiple nuclear alterations and centrosome amplification

**Figure 5:**
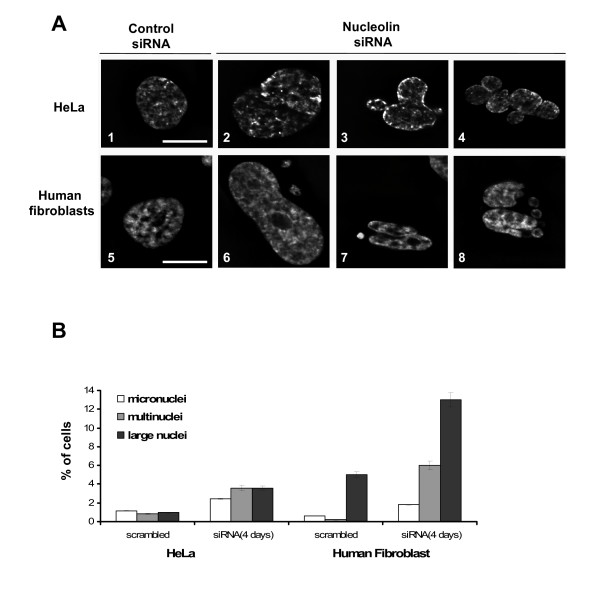
**Nucleolin depletion induces the formation of micronuclei and multinuclear cells**. A. Images of HeLa cells or human primary fibroblasts transfected for 4 days with scrambled siRNA #1 or with nucleolin siRNA mix #2 and #4 were counterstained with DAPI and visualized at the same magnification. Micronuclei are observed on A6 and A7, multinuclear cells are observed on A3, A4 and A8, large nuclei are observed on A2 and A6. Bar represents 10 μm. B. Table shows the percentage of multinuclear cells, and cells with large nuclei or micronuclei in HeLa cells or primary human fibroblasts, determined by cell counting under the microscope. n = 1740 for HeLa cells transfected with scrambled siRNA #1, n = 1380 for HeLa cells transfected with nucleolin siRNA mix, n = 1560 for fibroblasts transfected with scrambled siRNA #1, n = 1220 for fibroblasts transfected with nucleolin siRNA mix.

**Figure 6:**
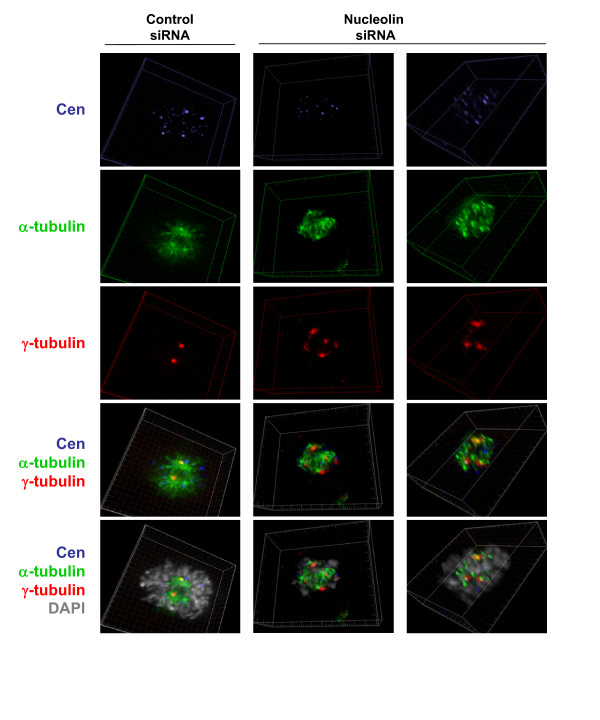
**Depletion of nucleolin induces hyper amplification of centrosome and multipolar mitotic spindle formation**. HeLa cells transfected for 4 days with control siRNA # 1 or with the nucleolin siRNA mix #2 and #4 were processed for immunofluorescence. Centromeres were stained with a CREST antiserum (CEN), microtubules with an α-tubulin antibody, centrosomal structures with a γ-tubulin antibody and DAPI was used to counterstained DNA. Views of 3D reconstructions are shown for each label and for merged labels as indicated.

Observation of nucleolin depleted cells also revealed a large increase in nuclear alterations (Figure 5A, B). In particular, the presence of cells with micronuclei or multiple nuclei was drastically increased in transfected cells. Compared to HeLa cells, this increase was even more pronounced in human primary fibroblasts, which exhibit almost no micronuclei or multinuclei in the population of cells transfected with scrambled siRNA. The presence of these defects could be the consequence of abnormal mitosis caused by amplified centrosomes [50]. To test the hypothesis that the depletion of nucleolin could lead to abnormalities in centrosome duplication, cells transfected with siRNA against nucleolin or control siRNA were stained with an anti-γ-tubulin antibody to detect the centrosomal structures, an anti-α-tubulin antibody to detect microtubules, a CREST serum (CEN) for the centromeres, and DAPI for DNA. As observed on 3D reconstructions of mitotic cells (Figure 6), more than 2 signals were consistently observed for γ-tubulin in siRNA transfected HeLa cells compared to 2 signals in control cells. Centrosome amplification in siRNA depleted mitotic cells was associated with the presence of a multipolar spindle as detected by α-tubulin staining (Figure 6).

## Discussion

Many reports suggest that nucleolin plays important roles in ribosome biogenesis, ranging from rDNA transcription to the assembly of the pre-ribosomal particles [11], and that this multifunctional protein may have a more general role in gene regulation through its involvement in chromatin structure and dynamics [35,37]. Nucleolin is highly expressed in tumors and actively dividing cells [51,52], and is often used as a marker for cell proliferation. Its ability to cooperate with Ras in a rat embryonic transformation assay [24], to repress translation of p53 mRNA [24], and to act as the target of the anti-proliferative GROs (G-rich oligonucleotides) [53,54], suggests that nucleolin could directly affect cell proliferation independently of its action in ribosome biogenesis.

To explore the role of nucleolin in cell proliferation, we used siRNA to repress the expression of nucleolin protein in HeLa cells and in cultured human primary fibroblasts. As soon as a significant decrease in the levels of nucleolin protein could be observed (2 fold decrease, 2 days after transfection), cells stopped proliferating (Figure 3), and a significant number of cells entered apoptosis (Figure 4). The slower growth of cells depleted in nucleolin is probably not the consequence of widespread alteration in gene expression. We performed protein labelling experiments in nucleolin depleted cells and did not observed major alterations in protein synthesis in these cells (data not shown). In addition, we have performed transcriptome analysis of HeLa and human fibroblast cells depleted in nucleolin. Surprisingly, only a limited number of genes (~0.1% of the genes present on the microarray) seem significantly affected (data not shown), suggesting that the slow growth of nucleolin depleted cells is not the result of a global effect of nucleolin on gene expression. Instead, changes in the expression of specific genes, such as p53 (Figure 4) might be responsible for slow growth and apoptosis. Future experiments will explore the different pathways that are activated upon nucleolin depletion.

One of the most drastic modifications of the ultra structure of the cell was the rearrangement of the nucleolar compartment (Figure 2). Fibrillar centers and dense fibrillar centers were delocalized to the periphery of the granular component, and a complete segregation of these compartments was also observed at later time points after siRNA transfection. This nucleolar reorganization had some resemblance to that obtained after the inhibition of rDNA transcription with low amount of actinomycin D [45-47], with injection of anti-UBF antibodies [49], or TIF-IA inactivation [55]. Indeed, we also observed that in nucleolin depleted cells, rDNA transcription was lower than in control cells (Figure 2D, E). However, unlike these earlier studies, we failed to observe a release of the major nucleolar proteins fibrillarin, UBF (figure 2B and 2C) or B23 (data not shown) in the nucleoplasm. Instead, UBF remained associated with the reorganized fibrillar component in interphase cells.

As previously described [49,55] this nucleolar disruption is accompanied by elevated levels of p53 protein (Figure 4). The increase in p53 levels was likely the consequence of the increased translation of p53 mRNA [24] and/or stabilization of p53 mRNA (Figure 4). As we have not directly tested if the interaction of HDM2 (mouse Double Minute 2 Human homolog) with p53 was altered in nucleolin deficient cells, we cannot exclude the possibility that the elevated level of p53 was also due to inhibition of HDM2, as previously reported [55,56].

One of the most remarkable consequences of nucleolin inactivation is the apparent blockage of cells in the G2 phase (Figure 3), and the significant increase in multinuclear cells and cells with micronuclei (Figure 5). By contrast, the number of cells in mitosis was drastically reduced in nucleolin depleted cells (Figure 3E, Additional file 5). Furthermore, in situ hybridization with a probe specific to the pericentromeres of chromosome 10 did not detect any significant increase in tetraploid cells, suggesting that cells are probably arrested in G2. This phenotype is quite different from that obtained upon inactivation of TIF-IA [55], but similar to that obtained upon inactivation of B23 [39]. Furthermore, as with the inactivation of B23, we also observed an alteration in the control of centrosome number in cells expressing low levels of nucleolin (Figure 6). Interestingly, it was also recently reported that nucleolin was required for chromosome congression and the maintenance of mitotic spindle integrity [57], suggesting that nucleolin may have a more general role during mitosis.

It is also striking that nucleolin and B23 share numerous homologous functions, despite the fact that they have different structures. Both interact with nucleic acids [5,11], shuttle between the nucleus and cytoplasm [58], and are involved in different aspects of ribosome biogenesis [10] and in chromatin regulation [34,35]. This report highlights other possible common properties of nucleolin and B23 in cell proliferation control and centrosome duplication.

It has been proposed that during mitosis, when the nuclear envelope breaks down, some B23 is delocalized from the nucleolus to centrosomes where it remains bound until it is phosphorylated by CDK2/cyclin E at mid-late G1 [41]. Some B23 phosphorylated on Thr^199 ^remained at the centrosome and seemed to be important for the interaction of the ROCK II kinase that also regulated centrosome duplication [59]. The regulated localization of B23 at the centrosomes could represent one of the licensing systems for centrosome duplication ensuring only one duplication per cell cycle [40]. Although nucleolin has been found associated with centrosomal structures in the proteomic analysis of human centrosomes [44], we have not been able to detect nucleolin in these structures by immunofluorescence using different antibodies. However, localization of B23 protein at the centrosomes is not easily detectable. Only a few B23 antibodies were able to detect B23 protein at the centrosomal structure [60], while conflicting data were obtained with GFP-B23 [42,60]. This may reflect an inaccessibility of the antibody to the protein, post-translational modifications or an alteration of the B23 structure when it was present at the centrosomes. Interestingly, nucleolin interacts with B23 [43,61], and several nucleolar proteins have been found associated with centrosomal structures in the proteomic characterization of the human centrosome [44]. This could suggest that a nucleolar protein complex could participate in the regulation of centrosome duplication. Future experiments should further explore this link between nucleolin and the regulation of centrosome duplication.

## Conclusion

In this report we uncovered a new role for nucleolin in cell proliferation and cell division. Unexpectedly we found that expression of nucleolin was required for a correct mitosis and controlled centrosome duplication. We expect that these findings will be useful to better understand not only the complex function of nucleolin in the cell but also the function of the nucleolar structure in other processes that ribosome biogenesis.

## Methods

### Cell culture and transfection

HeLa cells plated in Dulbecco's modified Eagle's medium (DMEM, Gibco BRL) supplemented with 10% (v/v) fetal calf serum (FCS, Life Technologies, Inc.) and 1% penicillin/streptomycin solution (Life Technologies, Inc.) were maintained at 37°C in 5% CO_2 _incubator. Primary human fibroblasts were grown in the same medium supplemented with 15% (v/v) fetal calf serum. Mixtures of functional siRNA specific for human nucleolin made by SMART pool technology, a control RISC-free siRNA were obtained from Dharmacon (Perbio Science France). Cells were plated at 1 × 10^5 ^in 6 well dishes and transfected with a final concentration of 100 nM siRNA by lipid transfection reagent DharmaFECT 1 from Dharmacon (Perbio Science France) according to the manufacturer's instructions.

### Cell proliferation and apoptosis assays

For FACS analysis, 1 × 10^6 ^cells of each sample were collected by trypsinization, fixed in 70% ethanol, washed in phosphate-buffered saline (PBS), resuspended in 1 ml of PBS containing 1 mg/ml RNase and 50 μg/ml propidium iodide, incubated in the dark for 30 min at room temperature, and analyzed at ~200 cells/sec on a Becton-Dickinson (Lincoln, NJ) FACScan. The percentages of cells in individual cell cycle phases were quantified with ModFit LD software.

A TUNEL assay (Promega) was used to identify apoptotic cells according to the supplier's recommendation. Briefly, cells were trypsinized, washed twice with PBS, and placed on glass slides using a Cytospin cytocentrifuge. Samples were fixed in 4% paraformaldehyde in PBS for 25 min at room temperature, and then permeabilized with 0.2% Triton X-100 (TX-100) in PBS for 10 min. After two washes with PBS cells were incubated with equilibration buffer for 10 min. Equilibration buffer was exchanged with TdT incubation mixture containing fluorescein-12-dUTP in the presence of terminal deoxynucleotidyl transferase (TdT) to label 3'-OH ends of fragmented DNA. The slides were incubated at 37°C for 60 min inside the humidified chamber in the dark. The reaction was stopped by immersing the slides in 2 × SSC for 15 min. After 3 washes in PBS the slides were mounted in Fluoromount G containing 400 ng/ml DAPI and analyzed with a fluorescence microscope (Leica DM IRB) using a standard fluorescein filter set. At least 400 cells were counted for each time point in two different experiments.

### Immunofluorescence and protein analysis

Cells were plated at 5 × 10^4 ^cells/well in 24 well dishes onto glass coverslips. Samples were washed with PBS, fixed in 4% paraformaldehyde in PBS for 5 min at room temperature, incubated in ice cold methanol for 20 min and then permeabilized with 0.1% Triton X-100 in PBS for 2 × 5 min. After two washes with PBS, nonspecific binding of antibodies was blocked by a 30 min incubation at room temperature with 10% fetal calf serum, 1% BSA and 0.1% TX-100 in PBS. After three washes with PBS, coverslips were incubated in primary antibodies at 37°C for 30 min as indicated below. After two washes in PBS/0.1% TX-100, coverslips were incubated with secondary antibodies coupled to Alexa dyes (A488, A568 and A647). After two more washes in PBS/0.1% TX-100, coverslips were washed in PBS, rinsed in ddH2O and briefly dipped in 100% EtOH. After a quick dry, coverslips were mounted on a slide with Fluoromount G (EMS) containing 400 ng/ml DAPI.

For protein analysis, cells were lysed in a 50 mM Tris-HCl, pH 7.5, 150 mM NaCl, 1% Nonidet P-40, 0.5% sodium deoxycholate, 0.1% sodium dodecyl sulfate [SDS] buffer, supplemented with complete protease cocktail inhibitor (Roche). 5 × 10^4 ^or 2 × 10^5 ^cells per slot were loaded onto a 10% SDS-polyacrylamide gel for electrophoresis and then transferred to Protran membranes (Schleicher & Schuell, Germany). Membranes were blocked in 5% milk, washed with PBST (PBS with 0.1% Tween), and incubated with the indicated primary antibodies. Following washing and incubation with horseradish peroxidase-conjugated anti-rabbit (Sigma) or anti-mouse (Sigma) antibody at 1:15000 and 1:5000 dilutions, respectively, in 5% milk, membranes were washed and detected by ECL (Amersham Biosciences) and exposed to x-ray film.

Antibodies for the detection of p53 (FL-393), cyclin A (H-432), PH3-S28 and cyclin B1 (GNS1) (all diluted 1:250, except 1:50 for PH3-S28) were obtained from Santa Cruz Biotechnology, Santa Cruz, CA. Nucleolin was detected with rabbit anti-nucleolin polyclonal antibody (A-134)(1:1000), rabbit polyclonal anti-B23 antibody (1:3000) was kindly gift from Dr M. Olson. A CREST serum was used at 1/4000 to detect centromeres and human auto-sera against fibrillarin and UBF were used at 1/100 (kind gifts from Dr. E. Tan, TSRI, La Jolla, USA). PH3S10 was detected with a rabbit polyclonal serum (kind gift from Dr. D. Allis, [48]) A directly coupled antibody against gamma-tubulin was used at 1/50 (Sigma).

### Microscopic image acquisition and visualisation

Images of cells were obtained with a motorized Zeiss Axioplan using a 63× objective lens (NA = 1.4), equipped with a CoolSNAP HQ CCD camera driven by Metamorph (Molecular Devices Corp., v. 6.3). Image stacks were collected at intervals of 0.2 μm in the z axis for all cells and processed using a 3D deconvolution procedure (Metamorph) using the measured PSF. Three to five sections containing focused signals were chosen for projection onto one plane for analysis (Figures 2B, C and 5A). Projections were further processed to generate digital masks using the magic wand and stroke tools in Adobe ^® ^Photoshop to ease visualization of as many as four fluorescent channels. Views of 3D reconstructions were generated using Amira using the complete z-stacks (Figure 6).

### Electron microscopy

Cells were fixed for 1 h at room temperature in a solution composed of 1.6% glutaraldehyde in 0.1 M Sorensen's buffer (pH 7.4). After three washes in the same buffer, the samples were acetylated according to Thiry et al. [62] before being embedded in Epon. Ultrathin sections mounted on copper grids were stained with uranyl acetate and lead citrate before examination with a Jeol CX 100 transmission electron microscope at 60 kV.

### RNA extraction and Real Time-PCR

Total RNA was extracted with RNeasy kit (Qiagen, France) and treated with DNase I (Promega). After extraction, the integrity of total RNA was examined on a 1.2% agarose gel containing 1 mg/ml ethidium bromide and quantified. 100 ng of total RNA were reverse-transcribed using random primers and 1^st ^Strand cDNA Synthesis Kit for RT-PCR (Roche Molecular Biochemicals). Real-time PCRs were performed with a Light Cycler 2.0 instrument (Roche Molecular Diagnostics) in Light Cycler capillaries using a commercially available master mix containing Taq DNA polymerase and SYBR-Green I deoxyribonucleoside triphosphates. Optimization reactions were first performed until the best primers, probe concentrations, and cycling conditions were established. After the addition of primers (final concentration: 0.5 μM), MgCl_2 _(4 mM) and template DNA to the master mix, 45 cycles of denaturation (95°C for 1 s), annealing (58°C for 10 s) and extension (72°C for 10 s) were performed. Cytoplasmic β-actin and18S rRNA were analyzed in parallel to each PCR, and the resulting actin and 18S rDNA measurements were used as internal standards for quantification of the specific transcripts as indicated.

## Authors' contributions

IU and CI carried out the set up of the siRNA system and most of the molecular and cellular analysis. IU, KM and FM carried out the immunofluorescence analysis assays. MT carried out the electron microscopy study. SS participated in the design of the experiments and helped to draft the manuscript. PB conceived the study, and participated in its design and coordination and drafts the manuscript. All authors read and approved the final manuscript.

## Supplementary Material

Additional file 1 Sequences of the differents siRNAs used in this study. Four (#1 to #4) siRNA directed against different domains of nucleolin (as indicated in the table) were synthesized and tested individually for nucleolin knock down. Two siRNA controls, scrambled (#1) or siGLORISC-free (from Dharmacon, sequence not available from the company) (#2) were also used in these experiments. Click here for file

Additional file 2 Efficiency of nucleolin down regulation with different siRNAs. A. Down regulation of nucleolin with four different individual siRNAs. HeLa cells were transfected with individual siRNA as indicated in material and methods. Lane 1, untransfected cells. Lane 2, scrambled siRNA #1. Lanes 3 to 6, individual siRNA. Lane 7, a mix of siRNA #2 and #4. Proteins were extracted 4 days after transfection and analyzed by western blot. The blot was successively probed with antibodies against nucleolin, tubulin and B23. B. Effect of a control siRNA #2 (SiGLO) on nucleolin expression. HeLa cells were transfected with a mix of siRNA #2 and #4 (lane 2) or with the control siRNA GLO (lane 3) and protein extracted 4 days after transfection. The blot was successively probed with antibodies against nucleolin and tubulin. Click here for file

Additional file 3 Effect of the different siRNAs on the cell cycle. HeLa cells untransfected or transfected for 4 days with control scrambled siRNA #1 or with individual siRNA against nucleolin #1 to #4, or with the mix of siRNA #2 and #4 were subjected to cell cycle analysis by flow cytometry. The numbers correspond to the percentage of cells in each cell cycle phase estimated with the Modfit software. Click here for file

Additional file 4 Effect of nucleolin depletion on BrdU incorporation and phosphor-H3 S^10 ^labeling. HeLa cells untransfected or transfected for 4 days with control siRNA #1 or with the siRNA mix #2 and #4 against nucleolin were subjected to BrdU incorporation. Microscopic scoring was performed on cells plated on coverslips and processed for immunofluorescence with antibodies against Phospho-H3 S^10 ^and BrdU. Click here for file

Additional file 5 Effect of nucleolin depletion on different mitotic markers. HeLa cells or primary human fibroblasts (HF) transfected with control siRNA #1 or with the siRNA mix # 2 and # 4 against nucleolin were used for different experiments as indicated in this table. Flow cytometry analysis allowed the estimation of cells exhibiting a G2/M DNA content. Mitosis was then evaluated by microscopic observation using three different criteria: DNA staining by DAPI, labeling with an anti-Phospho S^28 ^H3 or chromatin staining obtained with a cyclin B1 antibody. For each experiment, the total number of cells scored is indicated. Click here for file

Additional file 6 Apoptosis driven by nucleolin depletion cannot account for steady cell growth. In addition to growth curves presented on Figure 3A is represented a dashed line illustrating the growth of control cells submitted to apoptosis at a level comparable to nucleolin driven depletion. This simulated dashed curve is obtained by subtracting the number of apoptotic cells determined by tunnel assay (see Figure 4B) to the total number of cells for each time point, followed by a reevaluation of cell growth with this apoptotic-corrected cell number. Note that this simulated curve still illustrates a positive slop and is quite different from the steady growth curve obtained for cells transfected with siRNA against nucleolin (black squarres). Click here for file
