# Supplementary material for: Inactivation of nucleolin leads to nucleolar disruption, cell cycle arrest and defects in centrosome duplication
Source: BMC Mol Biol. 2007 Aug 10;8:66. doi: 10.1186/1471-2199-8-66 (PMC1976620; doi:10.1186/1471-2199-8-66)
Supplement: Additional file 1 — Sequences of the differents siRNAs used in this study. Four (#1 to #4) siRNA directed against different domains of nucleolin (as indicated in the table) were synthesized and tested individually for nucleolin knock down. Two siRNA controls, scrambled (#1) or siGLORISC-free (from Dharmacon, sequence not available from the company) (#2) were also used in these experiments. [file 1471-2199-8-66-S1.pdf]

siRNA	Protein domain	Nucleolin exon n°	Sequence 5' — 3'
**# 1**	RBD	6	UAAUUCAGGAGCAGAUUUGUU
**# 2**	RBD	13	UCCAAGGUAACUUUAUUUCUU
**# 3**	RGG	14	UCAAACUUCGUCUUCUUUCUU
**# 4**	Acidic strech & NLS	4 & 5	UUCUUUGACAGGCUCUUCCUU
**siRNA (scrambled) control 1**	-	-	GCUAGCUUUAUUCGUAUAUUA
**siRNA (siGLORISC-free) control 2**	-	-	Dharmacon, D-001600-01-05
