# Supplementary figures and images for: Inactivation of nucleolin leads to nucleolar disruption, cell cycle arrest and defects in centrosome duplication
Source: BMC Mol Biol. 2007 Aug 10;8:66. doi: 10.1186/1471-2199-8-66 (PMC1976620; doi:10.1186/1471-2199-8-66)

**A**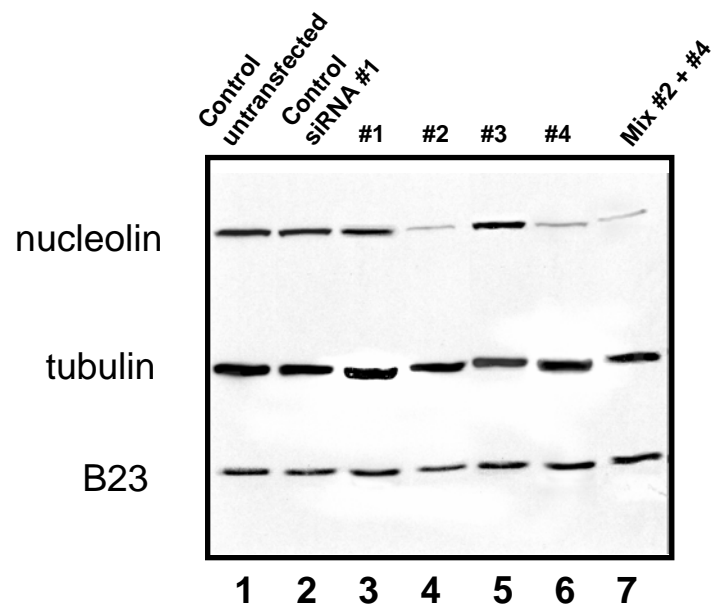**B**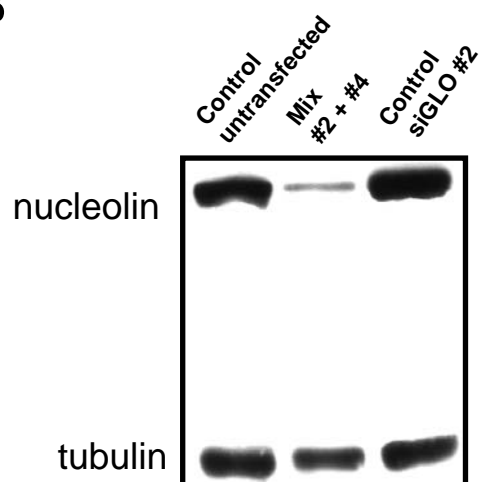

Supplement: Additional file 2 — Efficiency of nucleolin down regulation with different siRNAs. A. Down regulation of nucleolin with four different individual siRNAs. HeLa cells were transfected with individual siRNA as indicated in material and methods. Lane 1, untransfected cells. Lane 2, scrambled siRNA #1. Lanes 3 to 6, individual siRNA. Lane 7, a mix of siRNA #2 and #4. Proteins were extracted 4 days after transfection and analyzed by western blot. The blot was successively probed with antibodies against nucleolin, tubulin and B23. B. Effect of a control siRNA #2 (SiGLO) on nucleolin expression. HeLa cells were transfected with a mix of siRNA #2 and #4 (lane 2) or with the control siRNA GLO (lane 3) and protein extracted 4 days after transfection. The blot was successively probed with antibodies against nucleolin and tubulin. [file 1471-2199-8-66-S2.pdf]

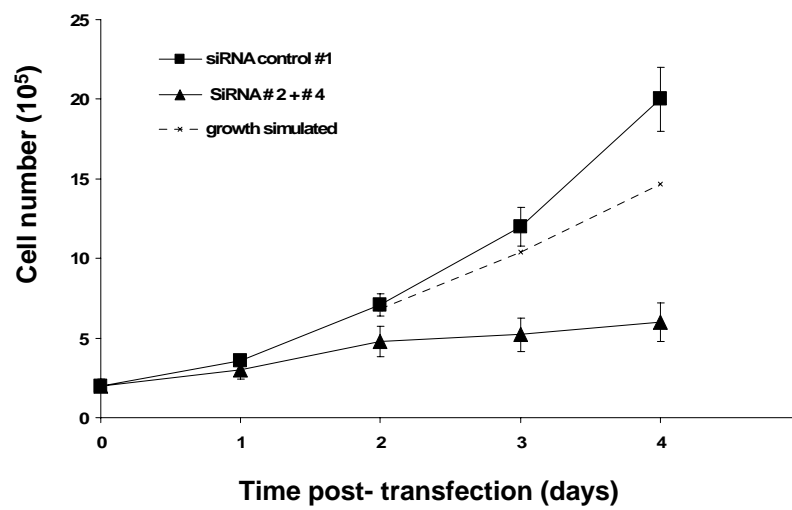

Supplement: Additional file 6 — Apoptosis driven by nucleolin depletion cannot account for steady cell growth. In addition to growth curves presented on Figure 3A is represented a dashed line illustrating the growth of control cells submitted to apoptosis at a level comparable to nucleolin driven depletion. This simulated dashed curve is obtained by subtracting the number of apoptotic cells determined by tunnel assay (see Figure 4B) to the total number of cells for each time point, followed by a reevaluation of cell growth with this apoptotic-corrected cell number. Note that this simulated curve still illustrates a positive slop and is quite different from the steady growth curve obtained for cells transfected with siRNA against nucleolin (black squarres). [file 1471-2199-8-66-S6.pdf]
