# Supplementary material for: Inactivation of nucleolin leads to nucleolar disruption, cell cycle arrest and defects in centrosome duplication
Source: BMC Mol Biol. 2007 Aug 10;8:66. doi: 10.1186/1471-2199-8-66 (PMC1976620; doi:10.1186/1471-2199-8-66)
Supplement: Additional file 3 — Effect of the different siRNAs on the cell cycle. HeLa cells untransfected or transfected for 4 days with control scrambled siRNA #1 or with individual siRNA against nucleolin #1 to #4, or with the mix of siRNA #2 and #4 were subjected to cell cycle analysis by flow cytometry. The numbers correspond to the percentage of cells in each cell cycle phase estimated with the Modfit software. [file 1471-2199-8-66-S3.pdf]

	Untransfected cells	siRNA control # 1	siRNA nucleolin # 1	siRNA nucleolin #2	siRNA nucleolin # 3	siRNA nucleolin #4	siRNA nucleolin Mix #2 and #4
**Sub G1**	3.2	2.03	6.7	13.79	4.4	8.23	10.4
**G0/G1**	53.4	56.08	46.6	34.37	55.3	40.59	37.9
**S**	36.5	33.98	35.4	34.53	35.2	34.98	35.4
**G2/M**	7.0	7.91	11.3	17.31	5.1	16.2	16.2
