# Supplementary material for: Inactivation of nucleolin leads to nucleolar disruption, cell cycle arrest and defects in centrosome duplication
Source: BMC Mol Biol. 2007 Aug 10;8:66. doi: 10.1186/1471-2199-8-66 (PMC1976620; doi:10.1186/1471-2199-8-66)
Supplement: Additional file 4 — Effect of nucleolin depletion on BrdU incorporation and phosphor-H3 S10 labeling. HeLa cells untransfected or transfected for 4 days with control siRNA #1 or with the siRNA mix #2 and #4 against nucleolin were subjected to BrdU incorporation. Microscopic scoring was performed on cells plated on coverslips and processed for immunofluorescence with antibodies against Phospho-H3 S10 and BrdU. [file 1471-2199-8-66-S4.pdf]

HeLa Cells	Phospho H3 S^{10}	BrdU incorporation	Number of cells
Control Untransfected cells	5.9%	36%	450
Control siRNA #1 (exp 1)	8%	29%	510
Control siRNA #1 (exp 2)	6.2%	27%	490
Nucleolin siRNA mix #2 and #4 (exp 1)	11.7%	31%	395
Nucleolin siRNA mix #2 and #4 (exp 2)	11.2%	29%	470
