# Supplementary material for: Inactivation of nucleolin leads to nucleolar disruption, cell cycle arrest and defects in centrosome duplication
Source: BMC Mol Biol. 2007 Aug 10;8:66. doi: 10.1186/1471-2199-8-66 (PMC1976620; doi:10.1186/1471-2199-8-66)
Supplement: Additional file 5 — Effect of nucleolin depletion on different mitotic markers. HeLa cells or primary human fibroblasts (HF) transfected with control siRNA #1 or with the siRNA mix # 2 and # 4 against nucleolin were used for different experiments as indicated in this table. Flow cytometry analysis allowed the estimation of cells exhibiting a G2/M DNA content. Mitosis was then evaluated by microscopic observation using three different criteria: DNA staining by DAPI, labeling with an anti-Phospho S28 H3 or chromatin staining obtained with a cyclin B1 antibody. For each experiment, the total number of cells scored is indicated. [file 1471-2199-8-66-S5.pdf]

	G2/M by FACS	Mitosis by DAPI	Number of cells	Mitosis by pH3S28	Number of cells	Mitosis by cyclin B1	Number of cells
**HeLa** siRNA control #1	7.9%	5.15%	1740	4.8%	797	8%	745
**HeLa siRNA (mix #2 and #4) 4 days**	17%	1.84%	1380	1.65%	623	3.2%	677
**HF** siRNA **control #1**	5.2%	3.7%	1560	3.5%	598	6.4%	490
**HF siRNA (mix #2 and #4) 4 days**	9.33%	0.3%	1200	0.3%	635	1.6%	398
